# Preimplantation Mouse Embryo Selection Guided by Light-Induced Dielectrophoresis

**DOI:** 10.1371/journal.pone.0010160

**Published:** 2010-04-13

**Authors:** Justin K. Valley, Paul Swinton, W. John Boscardin, Tom F. Lue, Paolo F. Rinaudo, Ming C. Wu, Maurice M. Garcia

**Affiliations:** 1 Berkeley Sensor & Actuator Center (BSAC), Electrical Engineering and Computer Sciences, University of California, Berkeley, California, United States of America; 2 Gladstone Transgenic Gene-Targeting Core Laboratory, University of California San Francisco, San Francisco, California, United States of America; 3 Department of Epidemiology and Biostatistics, University of California San Francisco, San Francisco, California, United States of America; 4 Department of Urology, University of California San Francisco, San Francisco, California, United States of America; 5 Division of Reproductive Endocrinology, Department of Obstetrics and Gynaecology, University of California San Francisco, San Francisco, California, United States of America; The University of Hong Kong, China

## Abstract

Selection of optimal quality embryos for *in vitro* fertilization (IVF) transfer is critical to successful live birth outcomes. Currently, embryos are chosen based on subjective assessment of morphologic developmental maturity. A non-invasive means to quantitatively measure an embryo's developmental maturity would reduce the variability introduced by the current standard. We present a method that exploits the scaling electrical properties of pre-transfer embryos to quantitatively discern embryo developmental maturity using light-induced dielectrophoresis (DEP). We show that an embryo's DEP response is highly correlated with its developmental stage. Uniquely, this technique allows one to select, in sequence and under blinded conditions, the most developmentally mature embryos among a mixed cohort of morphologically indistinguishable embryos cultured in optimized and sub-optimal culture media. Following assay, embryos continue to develop normally *in vitro*. Light-induced dielectrophoresis provides a non-invasive, quantitative, and reproducible means to select embryos for applications including IVF transfer and embryonic stem cell harvest.

## Introduction

Human IVF is one of the greatest scientific advances of the twentieth century. Since the first successful report of an IVF live birth in 1978[1], IVF has provided fertility to countless people previously considered infertile due to idiopathic causes, the natural aging process, anatomic abnormalities, and even the absence of sperm or eggs. Furthermore, IVF allowed human embryo development to be studied in real-time, beginning at the earliest stages of development. Additionally, IVF and related techniques, such as *in-vitro* culture, have made human embryonic stem cell research and therapies possible. The use of IVF has increased dramatically in the last 3 decades. In the U.S. today, 1–3% of all births are achieved using *in-vitro* assisted reproductive techniques (ART)[2], [3], [4].

Despite its rapid rise, IVF is criticized for significant limitations in 3 critical domains: *success rate* (defined as live births per number of embryos transferred), *morbidity* (health risk to mother and fetus), and *cost* (to patient, and health-care system)[2], [3]. In 2007, the proportion of IVF cycles that resulted in a live birth varied between 8.9% to 39.9%, and likelihood of success decreased significantly after the fourth cycle[5]. Due to the relatively low success rate of IVF, an average of 2–3 embryos are typically transferred to the mother per cycle; this results in a high multiple-birth rate (up to 34.7% in women >35 years of age)[6]. A multiple-birth pregnancy is the single greatest source of morbidity and mortality to both mother and fetus[2], [7], as these are closely associated with prematurity, low birth weight, Caesarian section, and, for both mother and fetus, increased risk of prolonged hospital stay, disability, or death[7], [8]. In the U.S., IVF is not provided by most health insurance plans and the average *cost* of a single cycle for IVF today is $12,400[9]; the average number of cycles per live birth is >3 (2007 data)[10]. Poor outcomes with respect to these 3 domains (success rate, morbidity, and cost) are rooted, at least in part, to our inability to reliably predict which 1–2 embryos, produced in-vitro, is likeliest to result in a live birth following transfer to the uterus.

Today, selection of specific embryos for uterine transfer is based primarily on morphologic parameters; only those that appear the most developmentally mature are selected. This practice is based on the notion that, since all embryos are fertilized at approximately the same time, those that have developed the furthest at a given time point are likeliest to have the greatest developmental potential. However, it is now accepted that morphologic parameters are not an entirely reliable index of embryo quality, and, as a consequence, intense interest is focused in developing more reliable methods for embryo selection[11], [12]. The low success rate, high risk of morbidity and mortality, and high cost could all be improved significantly if a metric were available with which to reliably predict the viability of each individual embryo, *prior to* transfer. This would make it possible to transfer only the healthiest and fewest number of embryos (ideally only one), and, thereby, reduce the rate of multiple births without reducing pregnancy rates[12], [13].

Dielectrophoresis (DEP) has been suggested as a potentially useful assay to guide embryo selection for transfer[14]. DEP refers to the response of the induced dipole moments of particles due to the application of an external non-uniform electric field[15]. It is used as a non-invasive technique to manipulate a multitude of objects ranging from cells[16], [17], [18] to nanowires[19], [20]. The response of an object, such as a cell, to DEP is characterized by the real part of the Clausius-Mossotti (CM) factor. This is an effective electrical polarizability of the object relative to that of the surrounding medium. The CM factor takes into account all of the physical properties of the object and media. This CM factor can either be positive or negative in value (attractive or repulsive forces) depending on the relative admittances of the particle (cell) and media. Cells in different physiologic states possess distinctly different electrical properties, resulting in different DEP responses[21], [22]. Accordingly, DEP has been used to distinguish between live, dead, and non-viable cells[23], [24], [25], as well as between different cells types[26].

**Figure 1:**
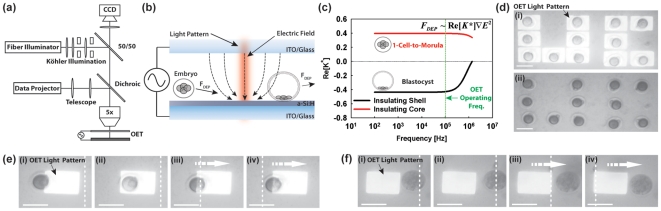
Overview of Light-induced Dielectrophoresis for Embryo Assessment. (a) Experimental Setup. Brightfield illumination and incident optical pattern (generated by data projector) are focused through a 5× objective onto OET substrate. Electrical bias is applied to the OET chip through a standard function generator. Viewing occurs through a topside CCD camera. (b) Schematic of OET device operation for embryonic assessment. Incident light interacts with a photosensitive layer of a-Si∶H. In conjunction with an externally applied bias, this causes the formation of electric field gradients (dotted lines) in the illuminated areas. These gradients result in a dielectrophoretic (DEP) force on embryos in the vicinity of the optical pattern. The response of the embryo can be either attractive (movement towards the light pattern) or repulsive (movement away from the light pattern) depending on the morphological state of the embryo. (c) Theoretical plot of Clausius-Mossetti (CM) factor for an insulating shell (blastocyst) and insulating core (1-cell-to-morula) versus frequency (Media conductivity: 10 mS/m). At the operating frequency (100 kHz), the model for the insulating shell (blastocyst) predicts a negative DEP response whereas it predicts a positive DEP (pDEP) response for the insulating core (1-cell-to-morula). (d) Demonstration of parallel manipulation of 1-cell embryos with optical pattern (i) and without (ii). Scale bar 100 µm. (e) Sequence of images of a 1-cell embryo undergoing pDEP response. White dotted line indicates a stationary point on the OET chip. Embryo is spontaneously attracted to light pattern (i)–(ii). Stage is moved relative to light pattern resulting in movement of embryo (arrow) (iii)–(iv). Scale bar 100 µm. (f) Sequence of images of a blastocyst undergoing nDEP response. White dotted line indicates a stationary point on the OET chip. Embryo is spontaneously repulsed from light pattern (i)–(ii). Stage is moved relative to light pattern resulting in movement of embryo (arrow) (iii)–(iv). Scale bar 100 µm.

In 2005, we reported a method termed Optoelectronic Tweezers (OET), which uses optical images to create DEP electrodes (light-induced dielectrophoresis)[27]. In the device, low intensity (<1 W/cm^2^) incoherent light interacts with a photosensitive substrate and, in conjunction with an externally applied electrical bias, creates localized DEP traps in the illuminated areas (**Figs. 1a** and **1b**). On-demand, parallel DEP trap generation is possible simply by altering the optical pattern. This technique affords many of the advantages of standard optical manipulation techniques (e.g., optical tweezers[28], plasmonic tweezers[29]), however using far less optical power (up to 10^5^× less[27]) as well removing the requirement of static electrodes used for more conventional DEP manipulation platforms[16], [20], [30], [31].

While the DEP response of oocytes and 1-cell (pre-cleavage) stage embryos has been studied[14],[32], the response of *post-cleavage* embryos to DEP, and, how such responses scale with developmental stage, has not been reported. Since pre-transfer embryo viability screening is performed primarily on post-cleavage stage embryos[33], it is essential to both understand, and be able to predict, the latter's response to DEP.

Given the multitude of structural changes that occur throughout embryo development from the 1-cell to expanded blastocyst stages, we hypothesized that an embryo's response to OET should change, in a predictable fashion, in parallel to developmental stage. Changes in morphology have been correlated to significant changes in the electrical properties of 1-cell to blastocyst stage embryos of various species[14], [34], [35]. This scaling of electrical properties can result in large fluctuations in the DEP response of pre-implantation stage embryos and, therefore, provide a *quantitative* means by which to assess embryo morphology and/or health (**Fig. 1c**).

Using a hybrid inbred mouse model and standard OET apparatus, in a 2-phase blinded study, we first determined how embryos, cultured in an optimized culture medium (KSOM+AA), respond to OET (DEP) at varying stages of development (1-cell, 2-cell, 4-to-16-cell/morula, and, early and late blastocyst stages). Next, to assess whether this technique could be used to guide embryo selection, we compared responses from embryos cultured in KSOM+AA to *morphologically identical* embryos cultured in a sub-optimal medium (M16). *In-vitro* culture in M16 yields, at all pre-implantation stages of development, a subset of embryos that are indistinguishable from ones cultured in KSOM+AA. However, M16 has been shown to sub-optimally sustain *in-vitro* embryo development, as compared to KSOM+AA, at all stages of development. This difference in quality between the two media is magnified as cultured embryos progress to later stages of development *in-vitro*. (For further discussion regarding the experimental design and choice of media see[36] and **Text S1**). Finally, as a preliminary effort to assess the safety of OET for embryos, the survival and continued *in-vitro* development of embryos following OET assay was analyzed.

## Results

**Figure 2:**
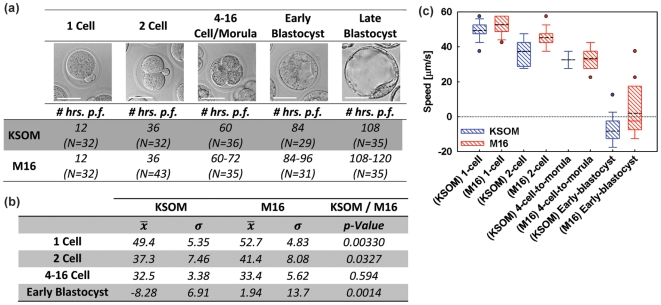
Experimental Results. (a) Summary of experimental groups showing representative images (4–16-cell/morula group shows only an 8-cell embryo), average number of hours post-fertilization (p.f.) each group was assayed at and cohort sizes (N). Note that the OET speed data for the late blastocyst group was not included in the following data analysis as many of the blastocysts were attached to the OET substrate making consistent speed measurements difficult. Scale bar 50 µm. (b) Table summarizing the mean and standard deviation of embryo velocities in both medias (all units are µm/s) as well as the *p*-Value between the two distributions. (c) Box plot showing maximum induced velocity in the OET device as a function of embryonic morphology (1-cell, 2-cell, 4-to-16-cell/morula, early blastocyst) and growth medium (KSOM+AA, M16). Black dotted line indicates mean. Note the transition from pDEP to nDEP as the embryos progress from the 1-cell stage to early blastocysts. Additionally, at all stages, except the 4-to-16-cell/morula stage, KSOM embryos exhibit a significantly less positive speed.

A total of 410 zygotes were harvested at the 1-cell stage and were divided equally into groups cultured in KSOM+AA and M16 medium. At the stages shown in **Fig. 2a**, cohorts of 29–43 embryos were taken from their respective culture medium, suspended in a low conductivity media (EP), and underwent OET assay (As defined in the **Materials and Methods** **Section**). The number of hours post fertilization that the embryo cohorts were assayed at each developmental group is tabulated in **Fig. 2a**. M16 cultured embryos generally required 6–12 hours of additional time in culture to reach equivalent *late* developmental stages, as embryos cultured in KSOM+AA. Maximum induced velocity (which is directly proportional to DEP force and, thus, the CM factor) was measured, using the manner described in Methods (See also, **Video S1**). Results are shown in **Figs. 2b** and **2c**. All embryos from both the KSOM+AA and M16 groups assayed at the 1-cell, 2-cell, and 4–16 cell/morula stages exhibited a positive DEP response (pDEP) to the assay OET field (attraction to the light pattern). Among *early blastocysts*, the majority of embryos cultured in either media exhibited a negative DEP (nDEP) response (*i.e.* repulsion from the light pattern). All *late blastocyst* and hatching embryos cultured in either medium also showed an nDEP response. Late blastocysts, and in particular, those that were partially hatched, were generally too adherent to the OET substrate to allow them to be moved long distances by the OET field. Thus, a reliable maximum OET-induced velocity could not be calculated for these groups, and they were excluded from further analysis.

Several trends are evident from the velocity data collected at each stage. For KSOM+AA embryos, the mean maximum induced velocity significantly decreased (became less positive) between each *successive* stage of development (*p<*0.006). Likewise, for M16 embryos, the mean maximum induced velocity also decreased significantly (*p*<0.0001) at each successive stage of development. Second, there were significant differences in mean OET-induced velocity between comparable KSOM+AA and M16 matched-pair groups. Among matched cohorts (morphologically indistinguishable embryos grown in either KSOM+AA or M16) at the 1-cell, 2-cell, and early blastocysts stages, those cultured in KSOM+AA exhibited a significantly less positive/more negative response to OET as compared to those from the M16 group (**Fig. 2b, c**). The group containing a mixture of 4–16-cell stage embryos was excluded from analysis *a prioi* due to the within-group morphologic heterogeneity. While induced velocities for this group paralleled the observed downward trend across all developmental stages, mean velocity for the 4–16-cell stage did not differ significantly (*p* = 0.59) between the 2 groups (**Fig. 2b,c**). Additionally, the variance among matched cohorts cultured in KSOM+AA and M16 and assayed at the 1-cell and 2-cell stages was not significantly different (*p* = 0.67 and *p* = 0.87, respectively). However, among embryos assayed at the 4-to-16-cell/morula and early-blastocyst stages, those cultured in KSOM+AA had significantly lower variance than matched cohorts cultured in M16 (**Fig. 2b**, *p*<0.0012 and *p*<0.015, respectively).

**Figure 3:**
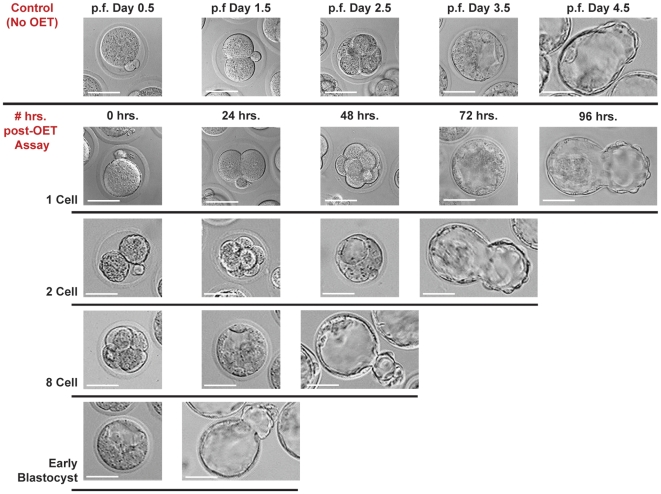
Viability of Embryos Post-assay. Representative pictures of embryos assayed in OET versus control group (not assayed in OET) at varying stages of development (1-cell, 2-cell, 8-cell, early blastocyst) and re-cultured in KSOM+AA media. Control group pictures are shown at 24 hour intervals post-fertilization (p.f.) starting at the 1-cell stage (p.f. Day 0.5) till the hatched blastocyst stage (p.f. Day 4.5). Post-OET Assay pictures were taken at 24 hr. intervals (following OET assay) until the embryos reached the hatched blastocyst stage. Nearly all (90–95%) assayed embryos, at all stages of development, progressed to the hatched phase. Scale bar 50 µm.

Immediately after OET assay, embryos appeared slightly contracted and granular (**Fig. 3**). This effect on embryo morphology appears to be attributable to the EP medium, rather than OET assay itself (**Fig. S1**). To better understand whether potential adverse effects on the embryos due to EP and OET were reversible, embryos that underwent initial OET assay (T = 0) at the 1-cell, 2-cell, 8-cell and early blastocyst stages, were recovered from the OET device, returned to incubation in KSOM+AA medium, and photographed every 24 hours thereafter. Ninety to 95% of embryos in each cohort continued to develop normally to the hatched blastocyst stage (**Fig. 3**). Long term exposure to EP media (5–24 hrs.) did result in eventual embryo death and the speeds of all embryos assayed after this long term culture were <5 µm/s (**Fig. S2** and **Text S1**).

Finally, to demonstrate the ease of parallel assessment/control of embryos using OET, a small cohort of 12 embryos cultured in KSOM+AA were retrieved from media at the 1-cell stage and underwent parallel OET manipulation to form the 2 letters “U” and “C” within the sorting chamber (**Fig. 1d**). All embryos responded positively to OET, and each was manipulated as shown in **Fig. 1e** and **Video S1**. Multiple light patterns (1 per embryo) were used to independently manipulate each embryo. After positioning each embryo, its associated light pattern could be left on (**Fig. 1d.i**), or, turned off (**Fig. 1d.ii**), as desired. Each embryo remained in place after the OET-induced DEP trap was turned off.

## Discussion

The observed progression from pDEP toward nDEP is likely due to changes in the electrical admittances of the developing embryo. At earlier stages of development (1-cell through 4-to-16-cell/morula), the embryo possesses a greater electrical admittance, relative to the surrounding medium. This is likely due to the highly conductive space between the zona pellucida and interiorly-located embryonic cells. This results in a positive CM factor, and, therefore, a pDEP response. However, starting at the early-blastocyst stage, the admittance of the embryos becomes smaller than that of the media, resulting in a negative valued CM factor, and, thus, an nDEP response. This is likely due to the formation of the trophoectoderm epithelium which electrically screens the highly conductive interior (blastocoele). Furthermore, as the embryo progresses from an early-stage to late-stage blastocyst, the trophectoderm's admittance decreases, resulting in an even larger nDEP response. This decrease (∼1000x) in admittance at the blastocyst stage has been confirmed by Benos *et al.* [34].

Given that OET can quantitatively distinguish embryos based on morphology, we hypothesized that the sensitivity of OET to detect such morphologic differences may be greater than current standard (purely observational) techniques. To assess this, the OET response of embryos cultured in either optimized culture medium (KSOM+AA) or sub-optimal medium (M16) was analyzed. KSOM+AA is the standard medium used for *in-vitro* mouse embryo culture models and has been systematically optimized over the years. M16, an “historic” medium formulation first reported in 1971, is deficient in several compounds that previous work has shown are necessary to sustain optimum *in-vitro* embryo development[36]. Because cohorts were matched for morphologic parameters and differed only with respect to which medium each was cultured in, any difference in embryo response to OET is likely attributed to developmental effects resulting from the culture medium.

We observed that, beginning as early as the 1-cell stage, mean OET response among embryos cultured in M16 was consistently and significantly different (*p*<0.05) than for matched cohorts cultured in KSOM+AA (**Fig. 2b,c**). Only the 4–16-cell cohort showed no significant difference, but, as mentioned, these groups contained subgroups of unequal numbers of embryos of varying morphology, and thus, these groups were *not* strictly comparable to one another.

If, as Biggers suggests, embryos are forced to “adapt” to abnormal conditions to survive (i.e. culture in M16), then, within any given cohort, some will adapt better than others, resulting in a spectrum of embryo viability and developmental potential[36]. Our results support this view: within all OET assayed cohorts, a range of OET responses was observed (**Fig. 2**). It is then reasonable to expect that, between the 2 culture groups at the same developmental stage, there will be some overlap. Though mean viability/developmental competence may differ significantly, a small subset of embryos cultured in the sub-optimal medium could be expected to have developmental potential comparable to sub-average embryos from the optimized medium group. Our results are again consistent with these assumptions: despite significant differences in mean OET response among matched cohorts cultured in both media, there was reasonable overlap in the actual OET response values of embryos from both media (**Fig. 2**). Furthermore, the variance in induced velocity among KSOM+AA cultured embryos decreased slightly (*p* = 0.53) and appeared to stabilize, whereas for matched cohorts cultured in M16, variance continued to increase through the early blastocyst stage (*p*≪0.0001). The upward trend in variance for M16 suggests that the longer the embryos are forced to adapt to a sub-optimal environment, the more the differences in viability and/or development are magnified.

Finally, the low conductivity (EP) medium in which the embryos are temporarily suspended in for OET assay deserves note. First, the medium conductivity must remain constant across all experiments in order to insure consistent results as the CM factor scales with media properties. Variation in conductivity of the final suspension was minimized through serial washing steps before each assay (See **Text S1** for additional discussion on medium conductivity and **Table S1** for individual cohort solution conductivities.). Second, the OET-compatible medium used here (EP) has not been optimized for compatibility with embryos. However, embryos which were assayed and then immediately returned to culture conditions in KSOM+AA (<30 min. exposure to EP) continued to develop at a normal rate with >90% reaching the late-blastocyst/hatched stage (**Fig. 3**). The latter suggests that minimizing exposure duration of each embryo to suspension media, and, use of a more embryo-compatible suspension medium, could preclude such potentially negative effects. Not surprisingly though, long term exposure (>5 hrs.) to EP media at room temperature consistently resulted in embryo death (**Fig. S2** and **Text S1**). Such observations are encouraging and warrant further and more rigorous studies to assess potential adverse effects on the embryos caused by OET assay.

How then could OET be used to guide embryo selection for IVF? Our results suggest that, for morphologically similar appearing embryos at any given stage, the embryo with the *most negative* response to OET is likeliest to be the most developmentally mature and/or viable, and should be selected for transfer. This approach is supported by both cross-developmental-stage, and, developmental-stage-matched, cross-medium comparisons (KSOM+AA and M16 cultured embryos). To date, it has simply been assumed that inferior embryo viability indices *in-vitro* predict inferior viability *post-transfer*. The proposed ability of OET to guide IVF embryo selection and improve outcome measures can only be validated by assessing post-transfer outcomes of embryos of mixed developmental potential selected by OET. However, the mere possibility that OET can non-invasively discriminate among embryos based on factors that cannot be seen by conventional means is exciting, and would have numerous possible applications including improved embryo selection for clinical and veterinary IVF, and, as a means to guide embryonic stem cell harvest.

## Materials and Methods

### Ethics Statement

Care and handling of all experimental animals used in this work were in accordance with University of California San Francisco's institutional animal care and use committee policies.

### Device Fabrication

A 6″ glass wafer with a 300 nm layer of sputtered indium tin oxide (ITO) (Thin Film Devices, USA) was coated with a 1 µm layer of hydrogenated amorphous silicon (a-Si∶H) deposited via plasma-enhanced chemical vapor deposition (PECVD) (100 sccm 10% SiH_4_∶Ar, 400 sccm Ar, 900 mTorr, 350°C, 200 W). The a-Si∶H coated ITO wafer, along with another 6″ ITO-coated glass wafer, was then diced into 2×2 cm chips with a dicing saw (ESEC 8003) forming the bottom and top OET substrates, respectively. The bottom OET substrate (a-Si∶H coated ITO) was then subjected to a brief oxygen plasma (51.1 sccm O_2_, 300 W, 1 min.) and placed in a solution of 2-[Methoxy(polyethyleneoxy)propyl]trimethoxysilane (Gelest Inc., USA) for 2 hours. The immersed chips were then rinsed in ethanol and air dried. This resulted in a thin layer of poly-ethylene glycol (PEG) on the surface of the bottom substrate which aided in reducing adherence of the embryos to the surface. Electrical contacts were made to the ITO on both the top and bottom substrate using an electrically conductive silver epoxy.

### OET Apparatus

A custom-built microscope was assembled and used for all experiments herein (**Fig. 1a**). The sample was placed on an XYZ micro manipulator (Newport, USA) connected to a mechanical stage drive (Newport LTA-HL and Newport ESP300-1NN111), which allowed the stage to be moved at a known rate. Viewing occurred from the topside via a 5× objective lens. Brightfield Köhler illumination was provided via a fiber illuminator (model OSL1, Thorlabs, USA) coupled through a 50/50 beam splitter. The optical patterns used for manipulation were formed using a commercial data projector (2400MP, Dell, USA) controlled by an external computer running commercial presentation software (Powerpoint 2003, Microsoft, USA). The images were focused onto the substrate by means of a telescope and long-pass dichoric mirror. Viewing and image capture occurred via a CCD camera (model XCD-X710CR, Sony, USA) connected to an external computer. Electrical bias was applied using a standard function generator (model 33220A, Agilent, USA).

### Embryo Harvest and *in vitro* Culture

Ovulation was induced by administering 5 IU PMS (IP) followed 48 hrs later by 5 IU HCG (IP) to 20 C57BL6 x DB2 F1 3–4 week old females (Charles River Labs, Worcester, MA.). Females were mated to 5 month old Male C57Bl6 mice (Harlan Laboratories, Inc). The following morning females were checked for the presence of a copulation plug. Embryos were then harvested from the oviducts of the plugged females. The cumulus cells where digested with 300 ug/ml Hyaluronidase (Sigma H4272) in M2 medium (Milipore, Billerica, MA). A total of 410 embryos were harvested and washed with M2, divided randomly into equal groups of 100, washed with respective pre warmed, C02 equilibrated culture medium and placed in 50 ul drops (33 embryos/drop) of pre warmed and C02 equilibrated medium under mineral Oil: KSOM+AA supplemented with amino acids (KSOM+AA) or M16 (Milipore, Billerica, MA.). Embryos were incubated at constant 37°C, 5%C02 (Fisher Scientific, USA). Embryo culture dishes were examined once daily beginning 8 hours from the midpoint of the dark cycle post-fertilization embryo development day (d) (d0.5), at the 1-cell stage. The above was performed on two consecutive days to have two developmental stages to evaluate on each day. The daily stages for these embryos are as follows: d1.5 (2-cell stage), d2.5 (4-cell to compacted 16-cell stage), d3.5 and d4.5 (early and late blastocyst stages, respectively). Embryos were examined and photographed under 200× and 800× microscope magnification using a Nikon Diaphot 200 Differential Interference Contrast (DIC) microscope connected to a CCD (COHU DSP 3600 Series, Poway, CA.). Embryos that failed to progress to the 2-cell stage, or appeared developmentally delayed by >24 hours at time evaluation of were removed from the culture dish and excluded from analysis.

### *In vitro* Development in KSOM+AA and M16

Ninety-percent of embryos cultured in either medium developed to the 2-cell stage on d1.5. All embryos that failed to progress to the 2-cell stage, and any abnormal or non-viable appearing embryos were excluded from the study and removed. On the morning of d2.5, many were noted to have already progressed to the 8 and 16-cell stages. To optimize statistical power for this group, we elected to assay mixtures of equal numbers of 4-cell, 8-cell, and compacted 16-cell embryos from each group. On the mornings of d3.5 and d4.5, approximately 70% of embryos cultured in KSOM+AA had progressed to the blastocyst stage, compared with only ∼35% of the M16 embryos. This difference in development rate between medium groups made it necessary to collect identical-appearing embryos from M16, for comparison to those in KSOM+AA, at a period of time 6–12 hours longer than required for the KSOM+AA group.

### Embryo Selection and Preparation for OET Assay

When, at time of primary examination (morning of p.f. days 0.5, 1.5, 2.5, 3.5 and 4.5), a minimum of 15 embryos had reached one of the given stages (1-cell, 2-cell, 4 to compacted 16-cell, early and late blastocyst), cohorts of 15–20 morphologically indistinguishable embryos were collected by aspiration micropipette and prepared for OET assay. To control for delayed maturation in either of the two media, embryos were collected only if a minimum of 15 embryos met criteria for collection (development to the target stage, with identical morphology within and across media groups for the given target stage. If fewer than 15 embryos met criteria for collection, none were collected and the entire medium-specific cohort was re-assessed every 4 hours thereafter, until a minimum of 15 embryos met criteria. Any abnormal and/or non-viable appearing embryos were excluded from the study and were removed at time of primary assessment every 24 hours.

Upon collection from medium, embryos were washed three times in Cytoporation (EP) Media T (Cytopulse Sciences, USA). EP medium is an isotonic OET-compatible buffer of minimal electric conductivity (10 mS/m.). Embryo cohorts were collected in a blinded fashion, suspended in 50–100 µL of EP medium, and placed onto the OET embryo sorting platform. The conductivity of the final solution containing each embryo cohort was measured. For embryos cultured in KSOM+AA, mean conductivity (at all stages) was 20.22±1.24 mS/m, and for embryos cultured in M16 (all stages) was 20.21±1.78 mS/m. Media conductivities at each stage of development for both KSOM+AA and M16 are tabulated in **Table S1**.

### OET Assay

The top OET substrate of the device was placed on top of the solution containing the embryos and separated from the bottom substrate by a 200 µm spacer. The device, now containing the embryos, was placed upon the manipulation stage and electrical bias was applied (20 Vppk, 100 kHz).

The DEP response and maximal DEP-induced velocity was then measured by projecting a rectangular light pattern onto the substrate (**Fig. 1e,f**, **Video S1**). The light pattern was positioned such that the leading edge of the light pattern was coincident with the outer edge of the embryo. The stage was then translated at varying speeds to extract the maximum speed at which the embryo could be moved by the adjacent light pattern. A positive dielectrophoretic (pDEP) response was defined when the embryo was attracted towards the center of the light pattern when the light pattern was brought near the embryo (**Fig. 1e**, **Video S1**). The fastest pDEP speed was defined as the maximum stage speed (light pattern) at which the embryo could still stay within the confines of the light pattern (i.e. the minimum speed at which the light pattern could no longer trap the embryo). pDEP speeds are annotated as a positive number. A negative dielectrophoretic (nDEP) response was recorded when the embryo was repulsed away from the edge of the light pattern when the light pattern was brought near the embryo (**Fig. 1f**, **Video S1**). The fastest nDEP speed was determined by finding the maximum stage (light pattern) speed at which the embryo could still stay outside the perimeter of the light pattern. nDEP speeds are annotated as a negative number.

### OET Assay of Embryos Subjected to Varying Times in EP Media

Cohorts of 20 randomly selected embryos from cohorts cultured in KSOM+AA were individually retrieved from the OET device immediately following assay at the 1-cell, 2-cell, 4-cell/morula, and early blastocyst stages (time, T = 0 hours). Each cohort was left in EP medium, at room temperature, for 24 hours (T = 24 hrs.), and thereafter, each was photographed (800× microscopy) and underwent repeat OET assay. An 8-cell group was also assayed at the 5 hr. mark (**Fig. S2**).

### Embryo Survival and Development in Culture After OET Assay

Twenty randomly selected embryos from each cohort cultured in KSOM+AA were extracted from the OET device after OET assay at the 1-cell, 2-cell, 8-cell, and early blastocyst stages. These were re-suspended in KSOM+AA and returned to incubation conditions. The embryos were then observed and photographed (800×) at 24 hr. intervals over 1–4 days (until the hatched blastocyst stage was reached) to assess the effects of OET on viability and development (**Fig. 3**).

### Medium Conductivity

The conductivity of the EP medium in which all batches of embryos were suspended during OET assay was measured (immediately before assay) using a hand-held conductivity meter (model B-173, Horiba, Japan).

### Statistical Analysis

All calculations were performed using the STATA 10 (College Station, TX.) statistical analysis software package. To test the difference in mean velocities, a two-sample Wilcoxon Rank-Sum test was performed. To test the difference in variance among groups, Levene's robust test for equality of variance was used.

## Supporting Information

Text S1 Supplementary Text.
(0.07 MB DOC)

Table S1 Final conductivity (mS/m) of each embryo group immediately prior to OET assay. Overall KSOM+AA, conductivity (at all stages) was 20.22±1.24 mS/m, and for embryos cultured in M16 (all stages) was 20.21±1.78 mS/m.
(0.07 MB DOC)

Figure S1 Effects of Assay Media. Representative pictures of 2-cell embryos after culture in KSOM (a), exposure to EP medium for 30 minutes (b), and assessment in OET while in EP medium (c). Scale bar 50 µm.
(0.35 MB PDF)

Figure S2 Effects of Assay Media on OET Speed. Maximum OET speed of 8-cell embryos and pictures after placement in EP media at 0 hrs. (a), 5 hrs. (b), and 24 hrs. (c). Cells within embryos undergo apoptosis after 5 hrs. and speed decreases monotonically to zero as time of incubation increases. Scale bar 50 µm.
(0.13 MB PDF)

Video S1 Video shows pDEP response in OET device of a of embryo at 1-cell stage, nDEP response in OET device of embryo at late blastocyst, and parallel manipulation of embryos within OET device.
(5.08 MB MP4)
