# Supplementary material for: Preimplantation Mouse Embryo Selection Guided by Light-Induced Dielectrophoresis
Source: PLoS One. 2010 Apr 13;5(4):e10160. doi: 10.1371/journal.pone.0010160 (PMC2854157; doi:10.1371/journal.pone.0010160)
Supplement: Text S1 — Supplementary Text. (0.07 MB DOC) [file pone.0010160.s001.doc]

**Supplementary Information Text:**

*Note on Long-term Exposure to EP Media*

To investigate the effects of long term exposure to the EP media used for OET assay, cohorts of 20 embryos cultured in KSOM+AA were assayed and extracted from the OET device at the 1-cell, 2-cell, 8-cell, and early blastocyst stages (Time, T=0 hrs.). After 24 hours, the maximum OET induced velocity for all embryos, at all stages assayed, had fallen to <5 μm/sec. One cohort of embryos (8-cell) also underwent repeat OET assay 5 hours after initial assay (**Fig. S2**). Four of the 20 embryos in this group appeared to have undergone additional cell division to between the 8-cell and morula stage. At this early time-point, mean OET induced velocity was significantly lower than the cohort’s initial response at T=0 (*p*<0.001) and mean variance was significantly greater than upon initial OET assay at T=0 (*p<0.001).* Almost all embryos subjected to the EP media for >5 hrs. underwent apoptosis and subsequent death. This clearly demonstrates that EP media is not optimized for embryo development and exposure to it during OET assay needs to be minimized.

*Note on Selection M16 and KSOM+AA as Culture Media*

The embryo model used here is predicated on three key points: 1. Embryos cultured in a suboptimal medium are inherently different, less likely to survive *in-vitro,* and/or result in a live birth, as compared with embryos cultured in optimized medium (KSOM+AA); 2. At any pre-implantation stage of development, embryos cultured in optimized medium can be visibly indistinguishable from embryos cultured in suboptimal medium; and 3. Clinically, embryo selection for IVF continues to be guided primarily by morphologic (*visible*) parameters. It is essential to review how embryo culture medium quality is defined.

In 1993, Lawitts and Biggers reported the formulation for KSOM medium. Beginning with a base-medium comprised of elements known to be necessary for embryo survival *in-vitro*, they used a novel algorithm called *sequential simplex optimization* to identify what additional components, and at what relative concentrations, are necessary to allow *in-vitro* developed embryos to match *in-vivo* developed embryos, with respect to established viability parameters[1,2,3]. This formulation was later modified by Ho, Schultz *et al.* to KSOM+AA, after showing that the addition of select amino acids consistently improved *in-vitro* embryo development to the late blastocyst stage[1,2]. Reverse-transcription polymerase chain assay studies have shown that culture in KSOM+AA results in housekeeping-gene and apoptosis-regulation gene expression that approximates expression *in-vivo*[2].

M16 medium is deficient in several compounds that previous work has shown are necessary to sustain optimum *in-vitro* embryo development[1,2,4], and renders most out-bred mouse strain embryos subject to 2-cell block[1]. Furthermore, compared with control (*in-utero developed*) embryos, a significantly smaller number of embryos cultured in M16 develop to reach the blastocyst stage. Of those that do, development takes place at a slower rate and with a lower number of inner cell mass cells[5]. More recently, Kamjoo *et al.* showed that beginning at the early blastocyst stage, embryos cultured in M16 displayed significantly fewer inner cell mass cells, and greater apoptosis, as compared with embryos cultured in KSOM+AA[6]. Hardy *et al.* showed that when the developmental rate for embryos cultured in M16 and KSOM is compared, the relative difference in rate steadily widens between the 1-cell and early blastocyst stages[5].

The prevailing view is that blocks in development observed *in-vitro* are most likely caused by adverse culture conditions, such as constituent absence or imbalanced concentrations[7], and, that adverse culture conditions result in sub-optimal embryo development due to either impaired transmission of maternal genetic genes and/or gene-expression[8], or defective activation of the embryo’s genome[9]. Prior to the introduction of KSOM+AA in 1995, the effects of culture medium formulation on post-transfer outcomes were rarely analyzed. This may be because media of this era were clearly not yet optimized to yield embryos developmentally comparable to controls (embryos developed *in-vivo* before transfer to a surrogate uterus). One such study, from 1970, showed that embryos cultured in a medium very similar to M16 (modified Brinster’s Medium), resulted in pre- and post-transfer embryo quality, and live birth rates, which were significantly inferior compared with control embryos[10]. Shultz *et al* have shown that in pups resulting from *in-vitro* culture (as compared with *in-vivo* controls), culture medium can result in significant long-term alterations in behavior (e.g. anxiety, locomotor activity, and spatial memory)[11]. M16 remains commercially available today, but its use is limited to short-term culture at the 1 and 2 cell stages.

Given that KSOM+AA is the standard media used for most *in-vitro* mouse embryo models, it was a clear choice for the “higher quality” media used in this study. As discussed, M16 is well documented to be inferior to KSOM+AA even though it has been widely used and reported on in the past. As a result, M16 was chosen as the “poor” quality media in this study.

*Note on Medium Conductivity:*

The medium conductivity used must fall in between the low and high admittance states of the developing embryos. Media conductivities outside of this range will result in either a pDEP or nDEP response regardless of embryo morphology. Therefore, it can be presumed that optimized media conductivities for different strains of embryos will be necessary in order to produce the largest dichotomy in response. In the context of these experiments, it is important that the media conductivity remain relatively constant for all groups due the dependence of the DEP response on the electrical properties of the media. This sensitivity to media conductivity is most prevalent at the point where the two complex permittivities are nearly identical (i.e. where the developing embryo transitions from a pDEP response to a nDEP response). To maximize internal consistency and precision, medium conductivity must be carefully monitored. It is also important to note that only certain conductivities of media (~1mS/m – 100 mS/m) can be used in the OET device presented here due to the fact that the liquid layer is part of the electrical circuit pertaining to device operation. For higher liquid conductivities (~1 S/m), a different OET device has been developed[12]. However, as above, at these high conductivities the embryos are unlikely to exhibit the large full scale range of DEP responses observed here.

**References:**

1. Biggers JD (1998) Reflections on the culture of the preimplantation embryo. International Journal of Developmental Biology 42: 879-884.

2. Ho Y, Wigglesworth K, Eppig JJ, Schultz RM (1995) Preimplantation development of mouse embryos in KSOM: augmentation by amino acids and analysis of gene expression. Mol Reprod Dev 41: 232-238.

3. Lawitts JA, Biggers JD (1993) Culture of preimplantation embryos. Methods Enzymol 225: 153-164.

4. Erbach GT, Lawitts JA, Papaioannou VE, Biggers JD (1994) Differential growth of the mouse preimplantation embryo in chemically defined media. Biology of Reproduction 50: 1027-1033.

5. Devreker F, Hardy K (1997) Effects of glutamine and taurine on preimplantation development and cleavage of mouse embryos in vitro. Biol Reprod 57: 921-928.

6. Kamjoo M, Brison DR, Kimber SJ (2002) Apoptosis in the preimplantation mouse embryo: effect of strain difference and in vitro culture. Mol Reprod Dev 61: 67-77.

7. Lawitts JA, Biggers JD (1991) Overcoming the 2-cell block by modifying standard components in a mouse embryo culture medium. Biol Reprod 45: 245-251.

8. Goddard MJ, Pratt HP (1983) Control of events during early cleavage of the mouse embryo: an analysis of the '2-cell block'. J Embryol Exp Morphol 73: 111-133.

9. Robl JM, Lohse-Heideman JK, First NL (1988) Strain differences in early mouse embryo development in vitro: role of the nucleus. J Exp Zool 247: 251-256.

10. Bowman P, McLaren A (1970) Viability and growth of mouse embryos after in vitro culture and fusion. J Embryol Exp Morphol 23: 693-704.

11. Ecker DJ, Stein P, Xu Z, Williams CJ, Kopf GS, et al. (2004) Long-term effects of culture of preimplantation mouse embryos on behavior. Proc Natl Acad Sci U S A 101: 1595-1600.

12. Hsu H-Y, Ohta AT, Chiou P-Y, Jamshidi A, Neale SL, et al. (2009) Phototransistor-based optoelectronic tweezers for dynamic cell manipulation in cell culture media. Lab on a Chip DOI: 10.1039/b906593h.
