# Supplementary material for: Preimplantation Mouse Embryo Selection Guided by Light-Induced Dielectrophoresis
Source: PLoS One. 2010 Apr 13;5(4):e10160. doi: 10.1371/journal.pone.0010160 (PMC2854157; doi:10.1371/journal.pone.0010160)
Supplement: Table S1 — Final conductivity (mS/m) of each embryo group immediately prior to OET assay. Overall KSOM+AA, conductivity (at all stages) was 20.22±1.24 mS/m, and for embryos cultured in M16 (all stages) was 20.21±1.78 mS/m. (0.07 MB DOC) [file pone.0010160.s002.doc]

|  | **KSOM** | | **M16** | |
| --- | --- | --- | --- | --- |
|  | $\bar{x}$ | ***σ*** | $\bar{x}$ | ***σ*** |
| **1 Cell** | *19.4* | *0.05* | *19.8* | *<0.01* |
| **2 Cell** | *20.3* | *1.38* | *18.5* | *2.09* |
| **4 Cell to Morula** | *20.7* | *0.96* | *21.4* | *0.49* |
| **Early Blastocyst** | *20.6* | *1.59* | *21.6* | *0.80* |
