# Supplementary figures and images for: Preimplantation Mouse Embryo Selection Guided by Light-Induced Dielectrophoresis
Source: PLoS One. 2010 Apr 13;5(4):e10160. doi: 10.1371/journal.pone.0010160 (PMC2854157; doi:10.1371/journal.pone.0010160)

**(a) KSOM**

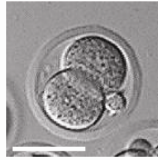

**(b) EP**

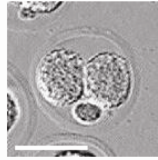

**(c) EP + OET**

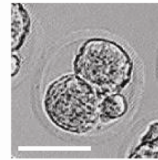

Supplement: Figure S1 — Effects of Assay Media. Representative pictures of 2-cell embryos after culture in KSOM (a), exposure to EP medium for 30 minutes (b), and assessment in OET while in EP medium (c). Scale bar 50 µm. (0.35 MB PDF) [file pone.0010160.s003.pdf]

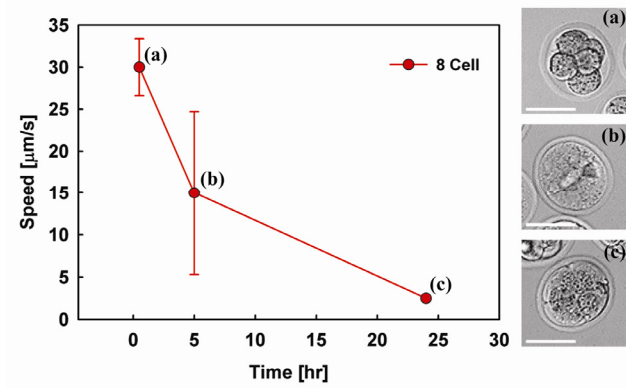

Supplement: Figure S2 — Effects of Assay Media on OET Speed. Maximum OET speed of 8-cell embryos and pictures after placement in EP media at 0 hrs. (a), 5 hrs. (b), and 24 hrs. (c). Cells within embryos undergo apoptosis after 5 hrs. and speed decreases monotonically to zero as time of incubation increases. Scale bar 50 µm. (0.13 MB PDF) [file pone.0010160.s004.pdf]
